# Open Questions about the Roles of DnaA, Related Proteins, and Hyperstructure Dynamics in the Cell Cycle

**DOI:** 10.3390/life13091890

**Published:** 2023-09-10

**Authors:** Masamichi Kohiyama, John Herrick, Vic Norris

**Affiliations:** 1 Institut Jacques Monod, Université Paris Cité, CNRS, 75013 Paris, France; masamichi.kohiyama@univ-paris-diderot.fr; 2 Independent Researcher, 3 rue des Jeûneurs, 75002 Paris, France; jhenryherrick@yahoo.fr; 3 CBSA UR 4312, University of Rouen Normandy, University of Caen Normandy, Normandy University, 76000 Rouen, France

**Keywords:** Charles E. Helmstetter Prize, *E. coli*, ribonucleotide reductase, sequestration, *oriC*, macromolecular crowding, differentiation, macromolecular synthesis operon, integrative suppression, L-form

## Abstract

The DnaA protein has long been considered to play the key role in the initiation of chromosome replication in modern bacteria. Many questions about this role, however, remain unanswered. Here, we raise these questions within a framework based on the dynamics of hyperstructures, alias large assemblies of molecules and macromolecules that perform a function. In these dynamics, hyperstructures can (1) emit and receive signals or (2) fuse and separate from one another. We ask whether the DnaA-based initiation hyperstructure acts as a logic gate receiving information from the membrane, the chromosome, and metabolism to trigger replication; we try to phrase some of these questions in terms of DNA supercoiling, strand opening, glycolytic enzymes, SeqA, ribonucleotide reductase, the macromolecular synthesis operon, post-translational modifications, and metabolic pools. Finally, we ask whether, underpinning the regulation of the cell cycle, there is a physico-chemical clock inherited from the first *protocells*, and whether this clock emits a single signal that triggers both chromosome replication and cell division.

## 1. Introduction

The coordination of cell growth and chromosome replication is achieved by mechanisms that are still being uncovered. One approach to investigating this coordination is genetics and, over the last half century, this has led to the isolation of conditional lethal mutants of cell division or DNA synthesis. As part of these investigations, in early 1960, Kohiyama started to isolate mutants of *Escherichia coli* K12 that fail to grow at a high temperature. With his collaborators, he found that nearly 1% of colonies obtained at 30 °C from a mutagenized culture failed to grow at 42 °C but, on examining each clone for DNA or protein syntheses and morphological changes after transfer to 42 °C, he found only a few mutants affected in DNA synthesis, with the majority being those defective in protein synthesis, such as valyl-sRNA synthetase [1], or in cell division, without identification of the mutated genes [2]. Isolation of temperature-sensitive (*ts*) mutants continued at the Pasteur Institute and the resulting strain collection has been beneficial to studies on the cell cycle such as the discovery of the FtsZ ring, which is essential for division [3], and to studies on metabolism such as those on the ribonucleotide reductase [4].

The first priority was the elucidation of the regulatory mechanism of chromosome replication as hypothesized in the Replicon Theory [5], according to which DNA replication starts from the genetically defined point (*oriC*) by the action of an initiator. Kohiyama, therefore, sought mutants that failed to initiate replication at high temperatures and found two [6]. These mutations were mapped to the same locus and the gene was called *dnaA* [7].

Further characterization of these *dnaA* mutants demonstrated a close connection between initiation of replication and cell cycle control: at a non-permissive temperature, a *dnaA* mutant temporarily stops dividing and forms filamentous cells [2]; division later resumes towards one end of the filament to produce normal-sized cells that lack DNA [8]. The fact that the size of these anucleate cells is relatively constant (but see [9]) is consistent with the idea that DnaA is involved, directly or indirectly, in the positioning of the division site.

In fact, the possibility that DnaA protein acts as a regulator of gene expression was raised by Hansen a few years after the isolation of the first mutant [10], and DnaA was subsequently shown to regulate many operons [11]. These observations, therefore, help make DnaA a candidate for the role of coordinator of the cell cycle.

**Figure 1:**
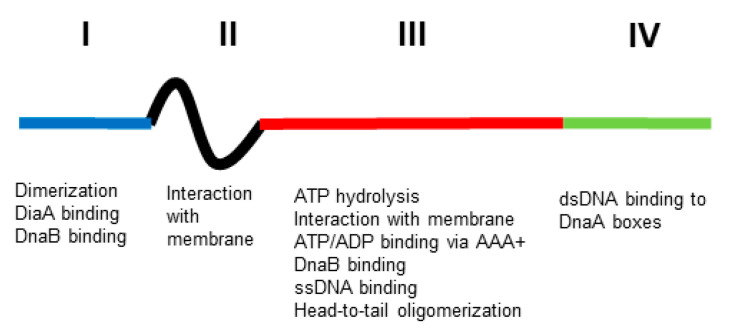
Functional structure of DnaA. The four domains (I, II, III and IV, blue, black, red and green, respectively) have different functions (see text for explanation). Not to scale.

To explore this proposal, it is essential to characterise the biochemical properties of the DnaA protein. A large part of this was done by Kornberg and his collaborators using genetic engineering only 20 years after the first isolation of a *dnaA* mutant [12]. They found that the DnaA protein is an ATPase possessing a high affinity for the replication origin (*oriC)* via DnaA boxes constituted of nine bases. The consequence of this interaction is the opening of *oriC*, which allows the insertion of DNA helicase into *oriC* in order to start DNA synthesis after the loading of DNA polymerase III. This interaction between DnaA and DnaA boxes seems to be important in most of the processes in which DnaA is involved [13,14]. DnaA has four domains [15,16] (Figure 1). Domain I binds both the helicase, DnaB, and the DiaA protein that may help link DnaA dimers and monomers; it also has a site for a low-affinity domain I–domain I interaction to generate dimers. Domain II is a linker. Domain III has an AAA+ motif that binds ATP or ADP; this binding of ATP leads to head-to-tail homo-oligomers in a helix; it also binds ssDNA and has a region that interacts with membrane. Domain IV is the dsDNA-binding domain that recognizes DnaA boxes (for references, see [11]).

Hyperstructures are large assemblies of molecules and macromolecules that have particular functions; they constitute a level of organization intermediate between the macromolecule and the cell. We have refrained from defining the concept too precisely because it is still “under development” and a prematurely narrow definition might limit its usefulness. In doing this, we adopt the principle of using a “generous Darwinian fog” [17].

Here, we raise questions that need to be addressed to clarify the roles of DnaA and related proteins in the cell cycle. We do this in the theoretical framework of hyperstructure dynamics and in the context of an origins-of-life scenario in which the early cells or *protocells* had a cell cycle regulated by a cellular clock or timer based on physical chemistry. We consider the possibility that DnaA—and the hyperstructures with which it is associated—are the heirs to the original clock and that they integrate different sorts of information in order to coordinate the cell cycle with the environment.

## 2. The Theoretical Framework for the Questions

**Figure 2:**
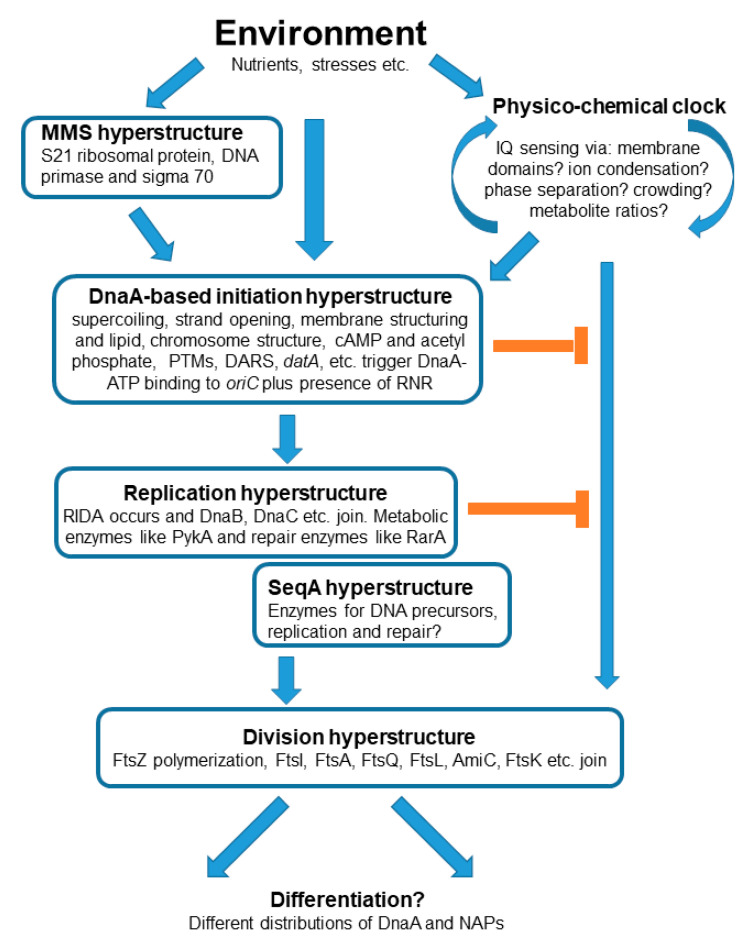
Control of the bacterial cell cycle by hyperstructures and DnaA. The boxes represent hyperstructures. A clock or timer based on physical–chemical processes provides the intensity and quantity (IQ) sensing needed for a successful cell cycle. This clock sends a signal to initiate both chromosome replication (via a DnaA-based initiation hyperstructure) and cell division. The initiation and replication hyperstructures can inhibit the cell division process (orange lines).

We propose that the precursors of cells, *alias* protocells, had a physico-chemical “clock” that emitted a single signal to trigger, simultaneously, the processes of both DNA replication and cell division. If so, it is conceivable that this clock still functions in modern cells but that this functioning now involves sophisticated macromolecules and the complex structures (hyperstructures) into which these macromolecules assemble in order to function. In this framework, we ask how the different hyperstructures involving DnaA and related proteins might be central to the operation of this clock. Along with its partners, the DnaA-based hyperstructure integrates environmental information via the structures of the membrane and chromosome, and via metabolites and ions (Figure 2). The output of this integration is the signal for the cell to make the transition from the non-replicating to the replicating state; the latter state ends with the division that produces daughter cells with possibly different phenotypes as modulated by DnaA. This integration has characteristics shared with actions typical of both AND and OR logic gates insofar as some, but not all, of the information integrated by the DnaA-based initiation hyperstructure is required for it to trigger replication.

## 3. Crowding, Phase Separation, and the Cell Cycle

Biomolecular condensates are a class of hyperstructures that play major roles in prokaryotic and eukaryotic physiology (for a review see [18]). They form because of phase separation, which is due to transient, low-affinity, cohesive interactions between their constituent polymers, whilst stereo-specific, macromolecular interactions only play a secondary role [19]. These interactions are determined by molecular and macromolecular crowding [20]. Different crowding agents can have different effects, as illustrated by the compaction and DNA binding of HU [21]. This paves the way for hyperstructures to respond in different ways in transducing information from inside and outside the cell. In the case of the initiation of chromosome replication, a low membrane occupancy is needed for the activation or rejuvenation of DnaA [22,23], whilst macromolecular crowding is required for the replication of *oriC* in vitro [24]; in the case of chromosome segregation, phase separation is proposed to underpin the segregation of newly replicated chromosomes [25]; in the case of cell division, macromolecular crowding is required for the formation of FtsZ droplets in vitro [26]. Fundamental questions here include: Are crowding and phase separation the primary determinants of hyperstructure dynamics? Do they determine the assembly and disassembly of hyperstructures? Do they control the fusion and fission of hyperstructures? In short, are they the essence of the clock that controls the cell cycle?

## 4. Is There a Chromosomal DnaA Hyperstructure?

The chromosomal *datA* site is a 1 kb region that contains binding sites for DnaA and that helps in its inactivation [27]. An excess of *datA* sites results in a delay to the initiation of replication, whereas the lack of *datA* results in extra initiations, and it was originally suggested that the binding of DnaA to newly duplicated *datA* during *oriC* sequestration could help prevent premature reinitiation when *oriC* is desequestered [27]. When *datA* is negatively supercoiled, DnaA-ATP oligomers are stabilised, and *datA*-IHF interactions and DnaA-ATP hydrolysis are promoted [28]. These results are consistent with a *datA*-based chromosomal hyperstructure helping regulate initiation. The two chromosomal intergenic regions, DnaA-reactivating sequence 1 (DARS1) and DnaA-reactivating sequence 2 (DARS2), each contain a cluster of DnaA binding sites; these sites promote regeneration of DnaA-ATP from DnaA-ADP by nucleotide exchange, and thereby help to promote the initiation of replication [29]. (Note that DnaA-ADP is mainly monomeric and unable to go from high-affinity binding sites to nucleate polymerisation at the low-affinity binding sites in the origin of replication.) The reactivation of DnaA by DARS2 is coordinated by the site-specific binding to DARS2 of IHF and Fis; this binding of IHF is temporally regulated during the cell cycle [30], as is the binding of Fis, which occurs specifically prior to initiation [31]. It is thought that DARS1 is mainly involved in maintaining the origin concentration, whereas DARS2 is also involved in maintaining single cell synchrony [32].

After initiation, the ATPase activity of DnaA is stimulated by the regulatory inactivation of the DnaA (RIDA) complex composed of the Hda protein interacting with the DNA-loaded β-clamp [33]. Recently, it has been found that only the disruption of RIDA has a major effect on initiation (since DARS and *datA* can compensate for one another) [34]. All of this raises the question of whether the inactivation of DnaA takes place within a chromosomal DnaA hyperstructure.

DnaA is also a sequence-specific transcriptional regulator. Such regulators bind to sites that are distributed on the chromosome with a periodicity consistent with a solenoidal-type organization [35]; this organization would bring together a regulator and its sites into a hyperstructure. If this hyperstructure does indeed exist, what is its relationship with the *datA* and the DARS sites? Do the above constitute separate hyperstructures and, if so, how do they interact? Or are they all part of an initiation hyperstructure?

## 5. Is There a Membrane DnaA Hyperstructure?

The involvement of the membrane in the initiation of replication has long been known [36,37,38], and it is tempting to speculate that the initiation hyperstructure may also contain acidic phospholipids such as cardiolipin, and even that the hyperstructure is physically associated with the bilayer itself, possibly in a fluid state. Indeed, a significant proportion of the DnaA (10% or more) in the cell is associated with the membrane [39,40]. In this association, both domain II and the hydrophobic domain III of DnaA are important [41]. Mutations and deletions affecting the membrane interaction sequence in domain III of DnaA restored growth to cells with a lowered content of acidic phospholipids [42]. DnaA association with the membrane may dissociate ADP from DnaA depending on the degree of protein crowding on the membrane [22,23]. Insertion into or association with the membrane is fundamental to *transertion* (the coupled transcription, translation, and insertion of proteins into a membrane) and to the existence of transertion hyperstructures [43,44,45]; this raises the question of whether a DnaA hyperstructure based on transertion might exist for part of the cell cycle and, moreover, whether such transertion might be important for the existence and/or operation of a chromosomal DnaA or other hyperstructures.

## 6. Does the Initiation Hyperstructure Contain Glycolytic Enzymes?

Metabolism is coupled to DNA synthesis through nutrient richness and growth rate in a variety of ways. One way this occurs is via (p)ppGpp [46], with the transcription of *dnaA* in *E. coli* [47] and the level of DnaA protein in *C. crescentus* [48] being lowered by (p)ppGpp. Another way is via a central carbon metabolism that, in *E. coli*, can: (1) promote DnaA to its active DnaA-ATP form and its binding to *oriC* by cAMP (a regulator of this part of metabolism) [49]; (2) suppress the defects of the *dnaA46* mutant by changes in pyruvate and acetate metabolism [50]; (3) inhibit DnaA conversion to DnaA-ATP and its binding to *oriC* (via acetylation of DnaA, with acetyl-CoA and acetyl-phosphate as donors) (for references see [51]). Evidence for the involvement of the central carbon metabolism in DNA replication in *Bacillus subtilis* includes: (1) subunits of pyruvate dehydrogenase (PdhC) and related enzymes bind the origin of replication region, DnaC and DnaG inhibit the initiation of replication; (2) mutations in the genes of central carbon metabolism suppress initiation and elongation defects in *dnaC*, *dnaG*, and *dnaE* mutants; (3) mutations in *gapA* (which encodes glyceraldehyde 3-phosphate dehydrogenase) perturb the metabolic control of replication; (4) pyruvate kinase (PykA) can both inhibit initiation and stimulate elongation via proposed interactions with DnaC, DnaG, and DnaE as modulated, perhaps, by phosphorylation [51,52].

Given that metabolic enzymes can exist as hyperstructures in their own right, a question that arises here is whether metabolic enzymes are also part of an initiation hyperstructure. And a related question is whether the metabolites themselves are directly part of initiation and/or replication hyperstructures, not just by binding to the constituent proteins (as in the case of DnaA and cAMP [49]) or by being used to modify the protein (as in the case of DnaA and acetyl-CoA), but also by binding directly to RNA or DNA. If metabolites were indeed to bind the origin region, this would make the connection with the *Ring World*, an origins-of-life scenario in which small, double-stranded DNA rings were selected firstly because they catalysed the reactions of central carbon metabolism [53].

## 7. Does the DnaA-Initiation Hyperstructure Contain SeqA?

*E. coli* avoids multiple reinitiations of chromosome replication by a sequestration mechanism that depends on the SeqA protein binding preferentially to newly replicated, hemi-methylated GATC sites, many of which are clustered in *oriC*. This sequestration, which involves the membrane, occurs only when *oriC* is hemi-methylated [54] and when SeqA, which has an affinity for the membrane, is present [55,56]; the result is an inhibition of initiation [57]. SeqA forms multimers and SeqA-DNA complexes can cover 100 kb of DNA and are close or integral to the replication hyperstructure(s), and have a bidirectional movement that differs from that of the origins (which goes to the poles) [58,59]. GATC sites are clustered not only in the *oriC* region but also in many genes involved in the replication and repair of DNA (such as *dnaA*, *dnaC*, *dnaE*, *gyrA*, *topA*, *hepA*, *lhr*, *parE*, *mukB*, *recB*, *recD*, and *uvrA*), as well as genes involved in the synthesis of the precursors of DNA (such as *nrdA*, *purA*, *purF*, *purL*, *pyrD* and *pyrI*), consistent, perhaps, with the presence of these genes and their products in a replication hyperstructure [60]. One related question is whether the DnaA-initiation hyperstructure in its earliest form contains SeqA and, reciprocally, a second question is whether the replication hyperstructure contains DnaA?

## 8. What Is the Relationship between Strand Opening and DnaA Binding?

Kornberg’s group first showed that DnaA opens *oriC* in vitro depending on the presence of ATP [61]. The opening of *oriC* was detected by P1 nuclease sensitivity in this case, and repeatedly shown by other techniques such as KMnO_4_ modification [62] and DMS footprinting [63]. *oriC* contains (1) the DnaA-ATP-Oligomerization Region (DOR), with twelve DnaA boxes, and (2) the neighbouring Duplex Unwinding Element (DUE), which contains three AT-rich 13-mer repeats along with DnaA binding motifs. DnaA-ATP assembles into a pentamer via its binding to DnaA boxes in one half of the DOR and, progressively, via its binding to the single-stranded motifs in the DUE, which stabilizes the unwound DUE. This unwinding is promoted by the nucleoid-associated protein (NAP), IHF, or indeed by HU [14]. Katayama’s group analysed, also by P1 nuclease sensitivity, the opening of M13 *oriC* DNA by DnaA in vitro at the DUE using various types of mutated *oriC* and IHF; the results obtained were consistent with those obtained in vivo [64]. That said, it is difficult to follow the kinetics of *oriC* opening with these techniques.

Strick’s group performed a single molecule analysis on DnaA–*oriC*(2kb) interaction using an optical magnetic tweezer to follow the rapid kinetics of double-stranded DNA opening. They observed formation of stable complexes between supposedly DnaA-ATP oligomers and *oriC* with different degrees of positive supercoiling. The formation of these complexes occurred using an *oriC* that lacked the DUE, raising the question of whether they were studying a non-canonical reaction. Other questions include why the kinetics of the complex formation was not studied, why the formation of the complex did not occur constantly [65], and whether DnaA was actually present in the complex. It should be pointed out that the use of optical magnetic tweezers is technically demanding: it requires the attachment of *oriC* DNA to a magnetic bead followed by the selection of intact *oriC*-containing beads (which are easily damaged and consequently in a minority).

Techniques based on minicircles of DNA facilitate the detection of fine-scale modifications to the DNA structure. Using an *oriC* minicircle of 641 bp with three negative supercoils, Landoulsi and Kohiyama found that around 80% of this substrate was positively twisted three times during incubation with DnaA and that the efficiency of unwinding was affected by the degree of negative superhelicity of the minicircle (three negative turns proved more effective in causing unwinding than two or four negative turns). Unwinding of this *oriC* minicircle by DnaA was verified by Bal31 sensitivity (rather than by P1 nuclease sensitivity), whilst the presence of DnaA on the unwound minicircle was confirmed by an anti-DnaA antiserum. The problem raised by this work is that the unwinding did not require ATP [66]. It should also be noted that the above work on *oriC* minicircles depends on a sophisticated technique that requires the formation of circles from a linear 641 bp *oriC* fragment that can only be achieved in a glass capillary after overnight incubation in the presence of DNA ligase and ethidium bromide, which introduces superhelicity; modification of the superhelicity of minicircles resulting from DnaA action is scored after Topo I treatment and is not directly measured.

This work raised the question of whether or not the ATP-dependent opening of *oriC* by DnaA (as demonstrated by P1 nuclease sensitivity) is the unique pathway for the initiation of replication. The fact that the mutant isolated first, *dnaA46*, which has lost the ATP binding site, can grow normally at a low temperature indicates the existence of an alternative pathway whereby *oriC* can fire without ATP. Consistent with this, the growth of *dnaA46* is more sensitive than the wild type to gyrase inhibitors [67], whilst the opening of *oriC* minicircles by DnaA is sensitive to negative supercoiling densities [66] (see above). Another explanation, offered by Kaguni’s group, is that the DnaA46 protein, with the aid of DnaK, can form a structure similar to that of DnaA-ATP [68]. Although no data are presently available from X-ray crystallography of the whole molecule of DnaA or from cryoEM analysis of DnaA-*oriC*, significant advances have been made by the Berger group using DnaA from *Aquifex aeolicus* along with the nonhydrolyzable ATP analog AMP-PCP [15], and by Katayama and collaborators using a combination of biochemistry and computer simulations to model the central part of the *oriC*-DnaA-IHF complex [16].

## 9. Does DnaA Participate in Differentiation?

In the strand segregation hypothesis, a coherent phenotypic diversity is generated by the segregation of certain hyperstructures with only one of the parental DNA strands [69]; candidate hyperstructures for such asymmetric segregation include those containing the NAPs and the topoisomerases. An asymmetric segregation of a chromosomal DnaA hyperstructure is another seductive possibility: could DnaA play a particular role in generating phenotypic diversity (e.g., in preparing a population to confront stresses via its role in modulating gene expression) or in connecting different phenotypes with different patterns of the cell cycle—or indeed both?

## 10. What Modifications Does DnaA Undergo and What Are Their Roles?

It has been proposed that a hyperstructure might be assembled if enzymes (such as protein kinases and acetyltransferases) and their NAP substrates were to associate with one another in a positive feedback loop in which, for example, the modification of an NAP by its cognate enzyme increases the probability of colocation of both the NAPs and the enzyme [69]. In line with this, the acetylation of a lysine residue (K178) prevents DnaA from binding to ATP and inhibits initiation, whilst the acetylation of another lysine residue (K243) also inhibits initiation but does not affect the ATP/ADP binding affinity of DnaA or the ability of DnaA to bind to the *dnaA* promoter region and to DARS1 [70].

DnaA binds cAMP with a Kd of a similar order to that with which it binds to ATP; indeed, the affinity of DnaA for cAMP is such that most of the cell’s DnaA should be bound to cAMP when the latter is present at the physiological concentration of 1 µM [49]. cAMP bound to DnaA is chased by ATP but not by ADP (note that there is only one cAMP binding site on the protein [49]). In vitro, cAMP stimulates DnaA binding to *oriC* and to DnaA sites elsewhere in the chromosome [49]; in vivo, the addition of cAMP to a *cya* mutant (which encodes the adenylate cyclase that catalyses the production of cAMP) increased the level of DnaA [71]. Significantly, despite DnaA’s stability in vivo, it has recently been shown to be degraded in vivo in ATP depletion conditions [72], and one possibility is that cAMP helps to both protect DnaA from degradation and regenerate DnaA-ATP from DnaA-ADP (by causing the release of the bound ADP). This raises the question of whether the state of the environment as reflected in a cAMP signal is transduced by the level of DnaA and by the DnaA-ATP: DnaA-ADP ratio into the expression of DnaA-regulated genes and cell cycle timing.

In the case of *Caulobacter crescentus*, the phosphorylation status of CtrA is central to cell cycle progress [73]. The many possible post-translational modifications to DnaA and to other proteins in the initiation and replication hyperstructures, therefore, include phosphorylation, and several other modifications, such as succinylation, methylation, proprionylation, malonylation, deamidation of asparagines, and glycosylation (for references see [69]). Another post-translational modification—and one that is largely ignored—is the covalent addition of poly-(R)-3-hydroxybutyrate (PHB) to proteins [74]; one proposed function of such addition to NAPs would be to regulate their interaction with nucleic acids [75]. An important question is, therefore, whether DnaA undergoes modifications like the addition of PHB and, if so, does such modification help the type of hyperstructure into which DnaA assembles?

## 11. Is DnaA a Controller of Chromosomal Copy Numbers Rather Than a Timer?

Fralick found that the timing of initiation and the number of replicating chromosomes per cell (and the DNA/mass ratio) could be varied independently of one another in a temperature-sensitive *dnaA(ts)* mutant grown at different temperatures. These results were interpreted as DnaA being an essential component of the “replication apparatus” but not itself being the signal that triggers initiation [76,77]. This interpretation would be consistent with the finding that the time of initiation is not advanced by a 50% increase in the concentration of DnaA-ATP, with the authors concluding that although DnaA protein is required for initiation of synchronous and well-timed replication cycles, the accumulation of DnaA-ATP does not control the time of initiation [78]. It should be noted that stopping the transcription of *dnaA* only led to a small increase in cell size, as DnaA was diluted by growth, whilst only disrupting RIDA had a major effect on initiation [34]. Finally, a mathematical model has recently been proposed that combines the titration- and activation-of-DnaA strategies to explain how initiation might be timed at fast and slow growth rates and to give both a precise volume per origin and a constant volume between initiations [79].

## 12. Does the MMS Operon Play an Important Role in Initiation?

The macromolecular synthesis (MMS) operon is highly conserved [80]; in *E. coli*, it comprises three genes: *rpsU,* which encodes the S21 ribosomal protein, *dnaG,* which encodes the DNA primase involved in the initiation of chromosome replication, and *rpoD,* which encodes the principal sigma subunit of RNA polymerase (sigma70, the “house-keeping” sigma). This operon is subject to a complex pattern of internal and external regulation in which it is possible to regulate each of its three genes independently of the others. In a series of investigations of heterogeneous responses to environmental stresses in *Listeria monocytogenes,* it was found that acid and other stresses primarily selected for *rpsU* variants, in some of which 116 genes were upregulated, mainly those controlled by the alternative stress sigma factor SigB; this leads to the hypothesis (1) that single amino acid substitutions in RpsU enable *L. monocytogenes* to switch between high fitness–low stress resistance and low fitness–high stress resistance and (2) that RpsU interacts with the stressosome, a stress-related hyperstructure responsible for integrating information about multiple environmental stresses and transmitting this as signals [81,82]. How might this relate to hyperstructure dynamics? If the MMS operon exists as a hyperstructure based on coupled transcription–translation, speculative hypotheses that might be entertained include an MMS hyperstructure being physically associated with an initiation/replication hyperstructure or a ribosomal hyperstructure; such association could then supply newly synthesized proteins directly to the appropriate hyperstructure (in the case of the primase, for lagging strand synthesis). Alternatively, the association between the MMS hyperstructure and another hyperstructure could result in the sequestering of newly synthesized proteins leading, in *E. coli*, for example, to a reduction in the level of sigma70 thereby favouring the other sigma factors and the emergence of a stress-adapted phenotype.

## 13. Does DnaA or a DnaA-Based Initiation Hyperstructure Also Trigger Division?

DNA replication is clearly coupled to cell division, insofar as signalling systems exist to prevent division when DNA has been damaged [83]. These include the SOS system that, when induced by DNA damage, produces the SulA/SfiA protein (along with forty other proteins) to interfere with the action of the key protein in cell division, FtsZ [84]. Using synchronised populations of *E. coli*, it was found that the levels of *ftsZ* mRNA increase at the time of initiation of replication [85,86]. That said, different results have also been obtained [87]. Significantly, the 2 min, or *dcw,* cluster of genes in *E. coli* contains three DnaA boxes upstream of *ftsZ* (within *ftsQA*) that were, however, not found to affect *ftsZ* expression in the conditions tested [86,88]; their role, therefore, remains an intriguing, open question. For example, could these boxes serve to connect physically, via a DnaA polymer, the 2 min transertion stage of the division hyperstructure with the initiation hyperstructure? In *B. subtilis*, DnaA binds the promoter region of *ftsL*, which encodes a key cell division protein [89], whilst, in *C. crescentus*, DnaA binds in vitro to the promoter region of *ftsZ*, which is believed to be part of the DnaA regulon that coordinates the initiation of DNA replication with cell cycle progression [90,91].

In *E. coli*, the putative coupling of DNA replication to cell division is also supported by the fact that several *ts* mutants affected in the initiation or elongation steps of DNA replication stop dividing normally at a non-permissive temperature and form filamentous cells that resemble those formed by a *thy* mutant during thymine starvation [92]. This cessation of division does not, however, necessarily mean that some aspect of the replication of the chromosome (including termination of replication) is responsible for the initiation of cell division. Indeed, after further cultivation of *ts* replication mutants at the non-permissive temperature, division resumes towards the ends of the filamentous cells to produce cells that lack chromosomal DNA; these *anucleate* cells are of almost normal size [92,93]. This production of anucleate cells occurs with *dnaA, dnaC*, *dnaG* (*parB*), and *dnaB ts* mutants and requires the absence of the inhibitor of division, SulA/SfiA, and a mutation in *ftsZ* (*sfiB*) [92]. How might this production occur?

It may be significant that the above anucleate cell production also requires cAMP (via either the activity of the wild type *cya* gene or an exogenous supply of cAMP) along with the cAMP receptor protein, CAP [92]. CAP regulates the transcription of over 100 genes in *E. coli*, including those in the *lac* operon. It is therefore conceivable that (1) there are major differences in the structure of the membrane in the presence and absence of cAMP and CAP and (2) these differences could affect transertion (e.g., via Lac permease) and, hence, the membrane domain dynamics that are proposed to time and position division [94,95]. If membrane dynamics do, indeed, underpin the regulation of the cell cycle at a fundamental level, it would make sense for proteins such as DnaA to respond to this fundamental system too, given that these sophisticated proteins presumably evolved some time after protocells had achieved some mastery over replication and division.

It could also be argued that it would have made sense for the earliest protocells to have had the same signalling mechanism leading to both DNA replication and cell division. This is because the RNA and/or DNA in these protocells was probably short and, in the *Ring World* scenario, in the form of a population of ds RNA/DNA rings, each of which catalysed a different reaction [53,96]; hence, the fundamental problem that protocells had to solve was not how to divide after replicating a long stretch of DNA, but rather how to proceed successfully through a complete cell cycle, which is a single decision. Norris has proposed that making this decision requires both intensity-sensing (does a cellular constituent risk becoming limiting for growth?) and quantity-sensing (is there enough material to make viable daughter cells?) [97]. Once a signalling mechanism had been adopted, it would be understandable if modern cells had been constrained to have retained the essence of this mechanism (even if overlain by the complex web of modern macromolecules). It turns out that there is some evidence, based on the relationship between the physical properties of the membrane and the distribution of the nucleoids, consistent with the idea that the initiation of replication and the initiation of division might indeed be triggered at the same time and, if so, logically by the same process (like transertion) [98]. Phospholipids are not just associated with the initiation hyperstructure but also with the division hyperstructure, and in *B. subtilis*, for example, most of the phospholipid synthases are located in the membrane part of the hyperstructure, which is enriched in cardiolipin and phosphatidylethanolamine. Another finding consistent with a close relationship between DNA replication and cell division is that an excess of DnaA (or an effective excess due to a deletion of *datA*) resulted in cell division in the absence of replication to generate anucleate cells [99]. In terms of hyperstructures, one explanation for the dependence of anucleate cell production on cAMP by *dna(ts)* mutants is that the membrane dynamics driving both replication and division hyperstructures are affected by cAMP. For example, cAMP not only affects replication via its binding to DnaA [49] but also affects division [100] via, we propose, the composition and structure of the membrane and cytoplasm. This is because cAMP is central to the induction or repression of many genes, including those in the *lac* operon, which contains a membrane protein, the Lac permease, that has preferences for the physical state and lipid composition of the membrane [101,102,103,104] and that affects its bending rigidity [105]. Given that a Lac transertion hyperstructure contains hundreds of macromolecules, the alteration of the membrane and cytoplasm by this cAMP-dependent hyperstructure could well affect division.

## 14. Do Variations in the Speed of the Elongation Step of DNA Replication Matter?

Oscillations in the speed of the replisome along the *E. coli* chromosome have been reported, and possibly explained as being due to the initiation of new replisomes slowing the progress of existing ones [106]. Temporal oscillations in the speed of the replisome have also been found by others in *E. coli*, *Vibrio cholerae,* and *B. subtilis,* with short pauses at ribosomal genes [107]. These oscillations also showed a time-dependent or bilateral symmetry about the origin, consistent with global variations in the availability of an element essential for replication (see below). Significant variations in the level of ATP between individual *E. coli* cells have been reported [108], whilst complex oscillatory variations in this level occur in individual cells during the cell cycle, with an average maximum of 2.4 mM and minimum of 1.2 mM [109]. Variations in the speed of replication in different places in the chromosome have been proposed to help determine the phenotype [110]. Could such variations be studied at the level of single cells? One technique that might be used is the *CIS* technique (for *Combing and Imaging by Secondary Ion Mass Spectrometry*), which can detect individual DNA fragments labelled in vivo with stable isotopes on the scale of a few hundred base pairs [111,112].

## 15. Does an Initiation Hyperstructure Sense DNA Supercoiling?

The supercoiling state of chromosomal DNA varies according to the growth phase and to extracellular stresses such as osmotic shock, heat, pH, and antibiotics [113,114,115]. It can also vary along the chromosome and can form a spatiotemporal gradient running from replication origin to terminus on both arms of the *E. coli* chromosome [116]. DNA gyrase has been proposed to act as a negative regulator of DnaA-dependent replication initiation from *oriC* in *B. subtilis,* since gyrase activity decreases DnaA association with *oriC* and inhibits replication initiation [117]. A deficiency of Topoisomerase I increases negative supercoiling, which results in the formation of transcription-associated RNA-DNA hybrids (R-loops), and DnaA- and *oriC*-independent constitutive stable DNA replication [118]. In other words, the initiation hyperstructure can take more than one form in response to different inputs such as supercoiling and the state of DnaA, thereby acting as a logic gate.

## 16. Is Ribonucleotide Reductase an Essential Constituent of the Initiation Hyperstructure?

The initiation and elongation steps of chromosome replication are tightly coordinated and mutually dependent in all organisms: the inhibition of initiation results in an increase in elongation rates and vice versa [119,120,121,122,123]. The observation of a negative correlation between initiation and elongation suggests that either directly or indirectly, initiation of DNA replication and elongation of DNA synthesis are interdependent. In eukaryotes, under physiological conditions, a clear negative correlation has been observed between replicon size (length of DNA replicated bidirectionally) and DNA replication fork rates, while inhibiting elongation at replication forks induces the activation of additional replication origins termed “dormant origins” [124]. Could this interrelationship between DNA replication initiation and elongation involve ribonucleoside diphosphate reductase (RNR), which supplies the deoxyribonucleotides (dNTPs) that are essential for replication? Could there be a relationship between the oscillations in ATP during cell growth [109], the oscillations in the speed of the replisome with its pauses at ribosomal genes [107], and the activity of RNR—indeed, could a need to divert ribonucleotides into dNTPs be one explanation for why there is no transcription during the S phase in eukaryotes?

The pool of available dNTPs is critical for successful replication since, with a defective supply, the DNA is likely to be damaged. Decreases in the size of the dNTP pool result in increases in the C period and *vice versa*, consistent with a major role for this pool in replication speed [125,126,127,128]. It would make no apparent sense then for initiation of replication to occur without the availability of this pool—and without the ability of RNR to supply dNTPs continuously at the right rate. Localisation of RNR is consistent with this enzyme being part of a replication hyperstructure [129,130] and, importantly, only a very small pool of dNTPs accumulates in the cell, which would allow for no more than one half minute of replication [131,132]. This would suggest the need for ribonucleotide reductase to be present and active at or near the replication forks both at the time of initiation and during elongation. 

The question then is whether RNR must be functioning for initiation to occur—and, possibly, functioning in the right place? Put differently, could RNR act as a sensor—or allow the initiation hyperstructure to act as a sensor—in order to couple metabolism and cell growth with replication? If so, could the very activity of RNR determine its presence in the initiation hyperstructure and the ability of this hyperstructure to trigger replication, as proposed for functioning-dependent structures [133]?

The rate of replication fork movement in all organisms depends on the activity of the enzyme RNR, which controls the level and balance of dNTP pool sizes during DNA synthesis (the S phase in eukaryotes and the C period in bacteria). The rate of replication fork movement also depends on a number of other factors, including the lagging strand DNA polymerase DnaE in *B. subtilis* [134] and the replication elongation factor DnaX in *E. coli* [135]. Consistent with an initiation–elongation regulatory circuit, it has been found in *E. coli* that DnaA regulates the *nrdAB* gene, which encodes RNR [136,137,138,139]. Low levels/concentrations of DnaA-ATP stimulate *nrdAB* expression (presumably prior to initiation), whereas high levels inhibit *nrdAB* expression (presumably at the time of initiation). Various studies have shown that DnaA-ATP modulates the level of *nrdAB* transcription and RNR activity, such that the active DnaA-ATP form of the protein correlates with both the number of replication forks and dNTP levels [138,139]. Genetic evidence for the existence of such a regulatory circuit has been established with the identification of suppressors of elongation mutants (*dnaX*2016) in *E. coli* that usually map to the *dnaA* gene in both *E. coli* and *B. subtilis* [135,140], while suppressors of the mutant *hda* gene, which overinitiates DNA replication, map to the *nrdAB* gene [141,142]. Suppressors of the *dnaAcos* mutant, which also overinitiates DNA replication, likewise map to the *nrdAB* locus [143,144].

As noted above, initiation in *E. coli* is a complex process involving the formation of a multi-component replication hyperstructure (composed of RNR, DnaA, and possibly SeqA and other factors) that activates initiation at a specific cell mass called the “initiation mass” [129]. The initiation mass is independent, it appears, of cell growth rate. How the cell “senses” the initiation mass, and therefore “knows” when to initiate chromosome duplication, has remained a mystery since the initiation mass concept was first introduced over fifty years ago. It is known, however, that neither DnaA, which controls the frequency of initiation, nor RNR, which controls the rate of DNA synthesis, appears to be the primary determinant of the timing of initiation or of the setting of the initiation mass [78,139] (see above Section 11: **Is DnaA a Controller of Chromosomal Copy Numbers Rather than a Timer?**)

Cells with reduced dNTP levels, however, initiate DNA replication earlier in the cell cycle (immediately after cell division) compared to wild type cells, but at a relatively larger cell size and hence at the same initiation mass [127]. This observation might suggest a link between the rate of elongation and the initiation mass itself, since DNA replication is coupled to cell growth, albeit by an unknown mechanism [134]. This raises an interesting question: does the rate of elongation, which is coupled to cell growth in the mother cell, set the initiation mass in the daughter cells, instead of the initiation mass setting the time of initiation in the daughter cell cycles? If so, then the rate of elongation, rather than the initiation mass itself, might be the decisive parameter that determines the major events driving the bacterial cell cycle. Rephrasing the question more precisely: is the initiation mass a passive consequence of the coupling between replication and growth or is it, as is commonly believed, an active cause of initiation and its timing in the cell cycle?

The independence of the initiation mass from the cell growth rate strongly suggests a metabolic link to the signalling of replication initiation, a link that remains poorly elucidated to date. In eukaryotes, DNA synthesis takes place during the reductive (biosynthetic) phase of the cell cycle and coincides with an abrupt rise in reactive oxygen species (ROS) at the G1 oxidative phase/ S phase transition, which suggests that the cellular metabolic state plays a role in signalling the timing of initiation at a critical cell physiology/mass, at least in eukaryotes [145,146,147]. As mentioned above (see above Section 6: **Does the Initiation Hyperstructure Contain Glycolytic Enzymes?**), in *B. subtilis*, a number of enzymes involved in metabolism have been shown to be associated with both replication initiation and elongation (specifically, the DnaC helicase, DnaG primase, and DnaE lagging strand polymerase), whilst in *E. coli*, carbon metabolism plays an important role in DNA replication fidelity and correlates with DNA synthesis [148,149,150].

It is tempting to speculate that levels and balances in metabolite pool sizes play a significant regulatory role in signalling and controlling important cell cycle processes, such as the accumulation of the initiation mass, the timing of the initiation of DNA replication, the rate of chromosome elongation, and cell division. Clearly, these events are tightly coordinated and co-regulated. One way is via post-translational modifications such as the acetylation of DnaA (see above Section 10: **What Modifications Does DnaA Undergo and What Are Their roles**?), and it may be significant that the acetylation of RNR in human cells results in the reduction of the dNTP pool and DNA replication fork stalling [151]. That said, questions concerning the role of metabolism in coordinating and regulating critical cell cycle functions have yet to be fully answered. It would be interesting, for example, to investigate how NADP(H):NAD+, ATP:ADP, and NTP:dNTP pool sizes co-vary during the cell cycle and whether or not they might play a role in determining the initiation mass, and, thus, prove informative in revealing the mysterious mechanism(s) by which the initiation mass appears to coordinate and control the major events of the cell cycle—if, in fact, it does.

## 17. Miscellaneous Questions

Eberle and collaborators performed a series of experiments that largely entailed shifting a growing culture of a *dnaA(ts)* strain (and sometimes a *dnaC(ts)* strain) to the non-permissive temperature for an hour and then returning that culture to the permissive temperature in the presence or absence of chloramphenicol; in the former case, this resulted in four to five initiation events, as opposed to just one in the latter case [152]. Only ten minutes of inhibition of protein synthesis were needed to produce these extra initiations [153]. Could seeing initiation in terms of hyperstructure dynamics help explain these results? For example, is it possible that the inhibition of protein synthesis, which would disrupt an MMS hyperstructure (or perturb a hyperstructure to which the MMS operon would normally contribute), would, therefore, inhibit an initiation hyperstructure? A complementary possibility is that the drop in temperature resulted in the decondensation of ions from *oriC* and associated proteins within a hyperstructure, leading to the opening of the strands and initiation [97].

As mentioned above (see Section 15 **Does an Initiation Hyperstructure Sense DNA Supercoiling?**), *E. coli* can grow despite the inactivation of *oriC* and *dnaA,* provided cells lack enzymes such as RNase H, which removes RNA-DNA hybrids in the form of R-loops [154]. This is because replication can be initiated at multiple ectopic *oriK* sites (for which a consensus sequence has yet to be defined) elsewhere on the chromosome [155,156]. It has been proposed that this “constitutive stable replication” may be a relic of the replication used by early cells [157]. Assuming that increasing transcription at an *oriK* increases the probability of replication, it is tempting to speculate that such coupling could provide an intensity-sensing mechanism to allow replication of DNA before it becomes limiting for growth [158]. That said, it is difficult to square this simple mechanism with the apparently normal timing of minichromosome replication in conditions in which chromosome replication itself is random (see below).

Eliasson and Nordstrom used an integratively suppressed strain to investigate minichromosome replication [159]. In this strain, the chromosomal *oriC* is inactive and replication occurs at random from a plasmid origin (P1); despite this, the rounds of replication of minichromosomes as seen using density shifts occurred at cell cycle intervals, consistent with a signal for initiation still being generated at the normal time [159]. The authors argued against an artefact due to a lengthening of the eclipse period—a period during which a newly replicated origin is refractory to a second initiation event [160]—but, rather, proposed that the minichromosome replication they observed was not being triggered by the process of chromosome replication; in other words, the system that normally triggers chromosome replication continued working even when chromosome replication was random [159]. This result, therefore, appears to call into question models based on the chromosome being an integral part of the cell cycle clock. Is it possible to explain the result by invoking the operation of a “primitive” physico-chemical clock based, for example, on hyperstructure dynamics? In particular, could the result be explained by the cyclically changing states of metabolic, non-equilibrium hyperstructures [97]? Could it even be based on some sort of long-term cellular memory based on hyperstructures, analogous to the memory of exposure to inducer conferred by the existence of a Lac hyperstructure that, once created by a level of inducer, maintains the capacity to metabolise lactose in the subsequent absence of this high level [161,162,163]? If such a memory depended on the segregation of hyperstructures with the DNA strands over the generations, it could give a distribution of growth rates and corresponding cell cycle periods in the population [164].

L-forms are bacteria that manage to grow in the absence of a peptidoglycan layer. They can be obtained by different methods and can have different styles of growth [165]. Division still occurs in *E. coli* L-forms even though FtsZ levels are fivefold lower than in the cells from which the L-forms are derived [166]; indeed, division can occur in a *B. subtilis* L-form in the absence of FtsZ [167], which gives a possible insight into the mechanism of division in early cells [168]. What, then, of DNA replication and its relationship to cell division? The high ratio in a *B. subtilis* L-form of the number of genomes (as detected by hybridisation) to the number of colony-forming units was attributed to a weaker coupling between chromosome replication and cell division [169]. A similarly high ratio was found in an L-form of *Listeria monocytogenes,* though it was noted that a third of the L-forms could not form colonies [170]; it was also found in this study that, for large L-form cells, those with a high concentration of DNA divided more frequently than those with a low concentration, which was interpreted as the high density of DNA in the former case contributing to the initiation of membrane perturbations and shape changes [170]. That said, it is important to note that the volume of L-form cells can be much greater than that of their walled parents, in which case the L-forms might contain less DNA per volume unit than parental cells. An *E. coli* L-form revealed a dependence on calcium concentrations in the growth medium, with optimum growth at 32 and 37 °C, in 0.1 or 1.0 mM Ca^2+^, respectively [171]; this is an intriguing result given the relationship between temperature and ion condensation [172] and the putative role for ion condensation in hyperstructure dynamics and cell cycle regulation [97]. Open questions include whether initiation in L-forms depends on DnaA and *oriC* and where DnaA is located.

## 18. Discussion

The principles of molecular biology have been to isolate and characterise gene products. The success of this reductionist approach has laid the foundations for the complementary, integrative approach based on physics and physical chemistry that shows how these products interact. This is the approach to the bacterial cell cycle that we have adopted here. Jun and collaborators recently proposed a variant of the initiation-titration model [173] that fully exploits the existence of two forms of DnaA, their interconversion, and the distribution of two types of binding sites on the chromosome that they validated using a physics-based approach [174]. Kleckner and collaborators proposed that, following the completion of “chromosomal and divisome-related events”, what they term a “progression control complex”—in other words, a type of hyperstructure—would form [175]; in combination with a mass increase, the changes in this complex would trigger cell division and the release of the terminus regions (for generation 1) along with licensing the subsequent triggering of replication via DnaA, etc. (for generation 2). In this hypothesis, the two events of cell division and nucleoid transition (which leads to initiation of replication) are independent of one another and could be the separate results of a common upstream event. Boye and Nordstrom argued for chromosome replication and cell division having their own, independent, cycles that are coupled by checkpoints to ensure the correct order of events, with replication and division cycles for *E. coli* and replication and mitotic cycles for *Schizosaccharomyces pombe* [176]. The independence of these cycles in bacteria is evidenced when the checkpoints fail: blocking cell division with penicillin does not block chromosome replication, whilst blocking replication in the absence of the SOS system does not (ultimately) block division. In eukaryotic cells, *S. pombe* cells can go through mitosis without a preceding S phase, whilst in meiosis, cells can go through two consecutive reductive cell divisions without intervening DNA replication. Boye, Nordstrom and others propose that these cycles operate in parallel and that they may even be initiated around the same time (for references see [176]). We subscribe to this view but adopt a different approach.

Our approach has been to ask what problems confront systems in general when they must adapt to an environment so as to profit from opportunities for growth and yet survive stresses: this balancing act is “life on the scales”, by which we mean that cells are constrained by selective pressures to balance apparently incompatible requirements (e.g., to both grow and not grow) and, hence, to find apparently incompatible solutions (e.g., to invest in both non-equilibrium structures and equilibrium structures) [97]. Successful adaptation requires the selection of regulatory criteria that include sensing when their components risk limiting their growth, sensing when they have enough material for reproducing, sensing when they are becoming too big, avoiding having networks that interfere with one another, and anticipating environmental changes by differentiating. In bacteria, these requirements are met via the cell cycle. To take the case of differentiation, for example, the two daughter cells that result from the cell cycle naturally have different phenotypes unless the species has been selected to prevent this from occurring. This is because bacteria have an abundance of circuits in which locally positive feedback and globally negative feedback are combined; consider, for example, two copies of a gene resulting from replication—one for each future daughter cell—with both copies competing for access to a limited number of RNA polymerases and with the copy being transcribed having a greater chance of continuing to be transcribed (put differently, this is a “rich get richer and the poor get poorer” situation). A similar argument can be made for differentiation in terms of hyperstructures, with each daughter getting a different set [43,69,164]; this leads to the question of whether the generation of daughter cells by the cell cycle actually corresponds to a spandrel [177]).

Once a regulatory system with many interactions between essential components has been constructed, it is well-nigh impossible to replace it completely. With this, and with the above criteria in mind, we have tried to formulate hypotheses for the regulation of the cell cycle that can be grounded in a plausible origins-of-life scenario [53,178,179]. Since this system evolved before the emergence of sophisticated macromolecules, it probably depended on the physical chemistry of the interactions of a host of simple molecules in the form of “composomes” [180], the putative ancestors of hyperstructures. This physical chemistry probably included phase separation, molecular crowding, membrane domain formation, ion condensation, and, in general, the mechanisms responsible for hyperstructure dynamics. In accord with Occam’s Razor, we speculate that the regulation of the “cell cycle” of the early cells was a single triggering event.

In the context of a physico-chemical approach based on hyperstructures, we have tried here to frame questions about the actors in the regulation of the cell cycle of modern bacteria. The principal actor in the initiation of chromosome replication is DnaA. The sorts of questions that, therefore, need answering include whether there are chromosomal hyperstructures that depend on DnaA binding to its different sites in the origin, in DARS, in *datA,* and elsewhere; whether there is a membrane hyperstructure that depends on DnaA interacting with lipids; and whether these proposed hyperstructures can form part of a single larger hyperstructure that has a trajectory based on membrane dynamics, DNA supercoiling, crowding, phase separation, etc. (see for example [25]). In this trajectory, the DnaA hyperstructure would initiate not only chromosome replication but also, perhaps, cell division. This hyperstructure might act as a logic gate and take into account: the state of transcription, translation, and replication as interpreted via the putative hyperstructure created by the MMS operon; the state of metabolism as interpreted via the presence of ribonucleotide reductase or via the binding of metabolites to hyperstructure constituents (like that of cAMP to DnaA) or via the putative hyperstructure created by glycolytic enzymes; the state of the chromosome as interpreted via hyperstructures created by supercoiling. The sorts of questions that need answering include “does the DnaA-initiation hyperstructure contain SeqA?”, “what is the relationship between strand opening and DnaA binding?”, and “what modifications does DnaA undergo and what are their roles?”. At a deeper level, questions also include “does DnaA participate in differentiation?” and “does DnaA or a DnaA-based initiation hyperstructure also trigger division?”.

At a still deeper level, the fundamental question is whether the initiation of cell cycle events involves a dialogue between separate hyperstructures (e.g., via the exchange of molecules and macromolecules) or whether this initiation involves a single hyperstructure undergoing changes in structure and composition (or both …). Answering this question may require the development of new techniques, for example by using *electro-optic fluorescence* microscopy [181], by combining *Secondary Ion Mass Spectrometry* (which allows 50 nm scale localization of stable isotopes and, hence, cellular activity) [182,183] and *toponomics* [184] (which allows 2 nm scale localization of 100 different proteins) so as to elucidate hyperstructure dynamics, and by revisiting often forgotten papers [152,154,159].
